# Spatial Frequency Discrimination: Effects of Age, Reward, and Practice

**DOI:** 10.1371/journal.pone.0169800

**Published:** 2017-01-30

**Authors:** Carlijn van den Boomen, Judith Carolien Peters

**Affiliations:** 1 Dept. of Developmental Psychology, Utrecht University, Heidelberglaan 1, Room H0.66, CS, Utrecht, The Netherlands; 2 Department of Cognitive Neuroscience, Faculty of Psychology and Neuroscience, Maastricht University, MD Maastricht, The Netherlands; 3 Netherlands Institute for Neuroscience, Institute of the Royal Netherlands Academy of Arts and Sciences (KNAW), Amsterdam, Netherlands; Durham University, UNITED KINGDOM

## Abstract

Social interaction starts with perception of the world around you. This study investigated two fundamental issues regarding the development of discrimination of higher spatial frequencies, which are important building blocks of perception. Firstly, it mapped the typical developmental trajectory of higher spatial frequency discrimination. Secondly, it developed and validated a novel design that could be applied to improve atypically developed vision. Specifically, this study examined the effect of age and reward on task performance, practice effects, and motivation (i.e., number of trials completed) in a higher spatial frequency (reference frequency: 6 cycles per degree) discrimination task. We measured discrimination thresholds in children aged between 7 to 12 years and adults (N = 135). Reward was manipulated by presenting either positive reinforcement or punishment. Results showed a decrease in discrimination thresholds with age, thus revealing that higher spatial frequency discrimination continues to develop after 12 years of age. This development continues longer than previously shown for discrimination of lower spatial frequencies. Moreover, thresholds decreased during the run, indicating that discrimination abilities improved. Reward did not affect performance or improvement. However, in an additional group of 5–6 year-olds (N = 28) punishments resulted in the completion of fewer trials compared to reinforcements. In both reward conditions children aged 5–6 years completed only a fourth or half of the run (64 to 128 out of 254 trials) and were not motivated to continue. The design thus needs further adaptation before it can be applied to this age group. Children aged 7–12 years and adults completed the run, suggesting that the design is successful and motivating for children aged 7–12 years. This study thus presents developmental differences in higher spatial frequency discrimination thresholds. Furthermore, it presents a design that can be used in future developmental studies that require multiple stimulus presentations such as visual perceptual learning.

## Introduction

Social and cognitive performance starts with perception of the world around you. Visual perception is immature at birth and refines until early puberty [1,2]. Unfortunately, visual perception is atypical in various populations. For instance, children that recovered from congenital cataract remain insensitive to very specific visual properties such as higher spatial frequencies [3,4]. Spatial frequency processing is of interest in the current study because it is an important building block of visual perception. For instance, it plays a crucial and changing role in face perception throughout development [5]. The spatial frequency of a grating is the number of light to dark cycles over an area, often described in the number of cycles per degree of visual angle. Higher spatial frequencies represent a higher number of cycles and relate to perception of local details. Lower spatial frequencies represent a lower number of cycles and relate to perception of coarse global information [5,6,7]. Development of higher spatial frequency perception, as all visual functions, depends on visual input. Therefore, children with atypical perception might benefit from increased input through visual perceptual learning. However, before we can turn to adapt atypical vision in children two steps need to be taken. The first is to gain full understanding of typical development of spatial frequency perception, which would serve as a standard for the successfulness of learned functions. Second, we need to develop a task that improves spatial frequency discrimination and is motivating for children, and thus can be used in visual perceptual learning research. The current study aimed to fulfill both these goals.

The development of spatial frequency perception is characterized by increases in the highest perceivable frequency at maximum luminance contrast (i.e. acuity) [8] and decreases in the luminance contrast at which a particular spatial frequency can be perceived (i.e. contrast sensitivity) [2]. Furthermore, there is an increase in discrimination abilities between two or more spatial frequencies at one contrast level [2]. Developmental trajectories for contrast sensitivity and discrimination differ for lower and higher spatial frequency information. Newborns can perceive lower spatial frequency gratings at high contrast levels but contrast sensitivity matures slowly up until 9–12 years of age. Sensitivity to higher spatial frequencies at high contrasts emerges during the first year of life followed by a rapid development. Many studies report maturation at 3–6 years, although some found small improvements until 9–12 years [2,9–12]. Neural processing of lower and higher spatial frequencies at high contrasts continues to develop until 9–10 years of age [13,14]. Less is known about the development of spatial frequency discrimination. Lower spatial frequency (reference frequency of 1 and 3 cycles per degree (cpd)) discrimination thresholds decrease rapidly between 5 and 7 years and continue to decrease slowly thereafter [15]. The developmental trajectory of discrimination thresholds of higher spatial frequencies could differ from that of lower ones. However, to our current knowledge the developmental trajectory of higher spatial frequency discrimination thresholds is unknown. To fill this knowledge gap, the current study mapped changes in higher spatial frequency discrimination thresholds across childhood.

The second goal of the study was to design an optimal task that can be implemented in visual perceptual learning experiments for children. Literature consistently reports successful improvement of perceptual skills in adults through visual perceptual learning designs (for a review, see [16]). These designs aim to improve a perceptual skill through multiple repetitions of a stimulus presentation. For instance, to improve discrimination of higher spatial frequency gratings participants perceive multiple trials containing such gratings. Typically, the design includes a behavioral response, such as the selection of a target grating. The spatial frequency difference between the gratings adapts to the participant’s performance. As a result, discrimination ability typically improves after multiple trials [16,17]. Although visual perceptual learning is proven to be successful in adults its methods cannot be generalized directly to children who differ from adults in perceptual and cognitive skills (for reviews see [2,18]). A main issue is the required attentional capacity to complete multiple trials. A couple of studies successfully applied visual perceptual learning on other visual functions in children [19,20], but did not investigate whether methods could be further optimized to increase participation duration and task performance.

A promising candidate for improvement of visual perceptual learning methods in children is increasing motivation through positive reward. Theoretical models predict that positive reward improves learning of basic visual information [21]. In children cognitive performance is better during positive than negative reward in younger (7–8 years) but less so in older children (11–13 years; [22]). Although these reports imply that positive reward plays an important role in performance this is not yet tested for basic visual processes in different age groups. For instance the study on discrimination of lower spatial frequencies applied both positive and negative reinforcements [15]. It is unknown whether the type of reward (positive versus negative) differently affects performance in basic visual task such as spatial frequency discrimination. Overall, the possibilities for visual perceptual learning in children are promising but knowledge on the effect of reward on performance helps future studies in creating age-directed and motivating training programs that are suitable for children.

The current study had two specific aims: 1) We studied developmental differences in discrimination thresholds of higher spatial frequency information, using an adaptive two alternative forced-choice discrimination task during presentation of higher spatial frequency grating stimuli. Specific interest was in developmental differences between children aged 5 to 12 years. Previous research [15] indicated that from 5 years of age onwards children produce reliable psychophysical thresholds in spatial frequency discrimination tasks. Furthermore, maturation of contrast sensitivity and neural processing of spatial frequency occurs between these ages. We therefore expected a decrease in discrimination thresholds with age. 2) The study investigated whether the type of reward affects task performance. Reward was manipulated by dividing participants in a positive reinforcement or positive punishment group. In the reinforcement group points were earned for correct answers, in the punishment group which points were lost for incorrect answers. Even though improving discrimination of many ranges of spatial frequencies would be beneficial to clinical populations, we investigated the effects of reward on discrimination of higher spatial frequencies. These frequencies are particularly impaired in children treated cataract [3–4] and drive emotion discrimination in 3–8 year old children [23]. Furthermore, higher spatial frequencies were chosen for the practical reason that we could combine the two aims within one study. Participants performed 254 test-trials in the discrimination task, lasting approximately 25 minutes. This large number of trials allowed us to look for effects of age and reward on three aspects of task performance. First, we studied overall higher spatial frequency discrimination thresholds. In addition, changes in the thresholds during the task indicated whether the design successfully induced an effect of practicing, which can be seen as a requirement for effects of learning. Finally, the number of trials participants completed indicated how motivated the child was. We hypothesized that positive reinforcement would lead to lower thresholds and larger decreases in threshold during the task compared to positive punishment.

## Materials and Methods

### Ethics statement

The research meets all applicable standards for ethics of experimentation and research integrity. Children recruited were visitors from a science museum. All caretakers gave written informed consent for their child’s participation in the study. Adults were recruited among students and employees of Utrecht University provided written informed consent. A local ethical committee of Utrecht University approved the experimental procedure according to the Declaration of Helsinki (2008). Reported data can be accessed via a request to the data handling committee. Please contact the corresponding author for details.

### Participants

A total of 135 participants were included in the data analyses of discrimination thresholds, the participants were divided into 4 age groups (7–8: N = 35; 9–10: N = 44; 11–12 years: N = 30; and adults: N = 27; see Table 1 for specifications). One additional child of 7–8 years did not complete the task and was therefore not included in the analyses. Another group of 5–6 year-olds participated in the experiment. However, since only 5 children completed the task they are not included in the threshold analyses. These children are only included in the analyses on motivation (number of completed trials). As such, these analyses included 165 participants (see Table 1). All included participants had no developmental disorders and normal or corrected-to-normal vision. An additional 16 participants were excluded due to developmental disorders (5–6 years: N = 2; 7–8 years: N = 1; 7–8 years: N = 3; 9–10 years: N = 9; adults: N = 1), and 7 due to visual abnormalities (equally divided across child age groups).

**Table 1:** Number of participants per age-group, gender, and reward type, separately for analyses on thresholds and number of trials included.

Group	5–6 year	7–8 year	9–10 year	11–12 year	adults
**Threshold analyses**					
Age in months (range)		97	117	140	309
		(81–107)	(107–130)	(132–153)	(239–389)
N total		35	44	30	27
N males / females		16 / 19	18 / 26	13 / 17	13/14
N reinforcement / punishment		16 / 19	24 / 20	13 / 17	13/14
**Number of trials analyses**					
Age in months (range)	73	97	117	140	309
	(59–83)	(81–107)	(107–130)	(132–153)	(239–389)
N total	28	36	44	30	27
N males / females	19 / 9	16 / 20	18 / 26	13 / 17	13/14
N reinforcement / punishment	15 / 13	17 / 19	24 / 20	13 / 17	13/14

### Apparatus and stimuli

#### Apparatus

The stimuli were presented on a Philips 240S4 24” monitor with a pixel resolution of 1920x1200 (1 pixel = 0.027 degrees) and a frame refresh rate of 60 Hz. The Gabor stimuli were created using Neurobs Presentation (version 14.6).

#### Stimuli

**Fig 1:**
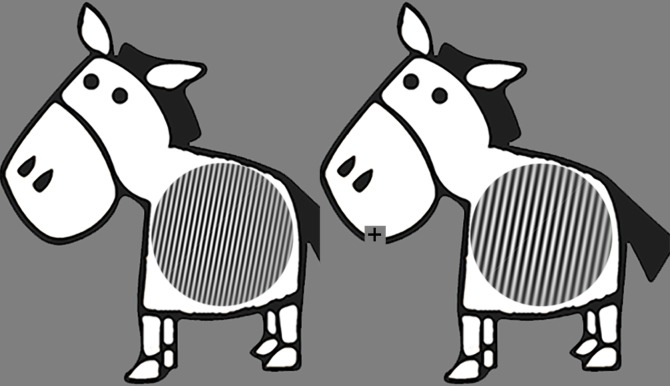
Examples of Gabor gratings forming zebras stripes, which are the stimuli presented to the participant. The right Gabor has a spatial frequency 33% lower than that of the reference left Gabor. Please note that stimuli are adapted for printing purposes, and that the spatial frequency depends on viewing distance. The present stimuli would have 6 cycles per degree (left) and 4 cycles per degree (right) if printed or displayed at 4.63 cm width and viewed at 57 cm distance. Refer to S1 Fig for an example of the original stimuli including a Gabor with 6 cpd if printed or displayed at 4.63 cm width and viewed at 57 cm distance.

In the first practice task stimuli consisted of two cartoons: Winnie the Pooh and Ernie, presented simultaneously at 8.1 degrees to the left and right of center. Stimuli of the other practice and experimental tasks were sinusoidal Gabor gratings that had a visual angle of 4.63 x 4.63 degrees, presented simultaneously at 5.95 degrees left and right of center. Gabor gratings were static, were tilted 10 degrees to the right, had a black-white color with high contrast-luminance, and a randomly jittered phase. Spatial frequency differed between Gabors. The reference Gabor grating had a spatial frequency of 6 cycles per degree (cpd; presented with equal probability at left or right side of center). The value of 6 cpd was chosen because it was the same or close to the higher spatial frequency gratings presented in previous developmental research on contrast sensitivity [10]: 6 cpd; [11]: 4.8 cpd) or electroencephalographic measures of spatial frequency processing ([13]: 6 cpd). Initially the spatial frequency of the other Gabor patch was 60% higher or lower (presented with equal probability to be higher or lower in all trials) compared to the reference Gabor. Importantly this difference in spatial frequency varied across trials. This was controlled by an adaptive staircase procedure targeting a discrimination accuracy of 84% [24]. Specifically, for every incorrect trial the difference increased by five percent, and for every four correct trials in a row the difference decreased by five percent. To make the task suitable for children a zebra was placed surrounding each Gabor grating, such that the Gabor grating appeared as the stripes of the zebra (Fig 1). All stimuli were presented on a grey background (RGB color: 127, 127, 127).

### Procedure

Experiments were performed in a quiet room at a science museum (children) or university building (adults). The room was illuminated by daylight without light being reflected by the monitor. If children were disturbed by noise in the museum they could wear noise-blocking headphones. The experimental procedures were explained to the participants and their parents, after which informed consent was obtained. Parents were permitted to remain in the testing room but were instructed not to assist the children with their task. For participants that could not read a parent or the experimenter was seated next to the child to read the feedback out loud. Participants were seated 57 cm from the monitor. The experiment consisted of 2 practice runs for participants aged 7 and older, and 3 practice runs for 5 to 6 year-olds. The experimental run was subsequently administered.

#### Practice run 1

The first practice run aimed to familiarize children with an experimental setting and with the response buttons. Children watched a Winnie the Pooh and Ernie cartoon. The participant had to indicate on which side Winnie the Pooh appeared. Written feedback on correctness was provided. This practice task consisted of two trials.

#### Practice run 2

Children aged 5 to 6 years then performed an additional practice run to familiarize them with the requirements of the experimental run. The practice run was added for this age-group, due to the fact that a pilot study revealed that the experimental run contained too many components for the child to understand at once. With the additional practice run these children could understand and perform the experimental run. Similarly to the experimental run children perceived Gabors appearing as zebras stripes and had to indicate which zebra had the most stripes (i.e. highest spatial frequency). Differently than in the experimental run, the stimuli were presented until the participant responded. Written feedback on correctness was provided. This practice run lasted until the experimenter was confident the child understood the task instructions, which was after approximately 5 trials.

#### Practice run 3

Subsequently, participants performed two trials of the experimental run to familiarize them with the stimulus sequence and task requirements.

#### Experimental run

The experimental run contained a 2 alternative forced-choice discrimination task consisting of 4 blocks of 64 trials each. Between blocks and halfway through each block participants could take a self-paced break. Each trial consisted of 2 Gabor gratings with different spatial frequencies appearing as zebras stripes. The participant had to indicate which zebra had the most and thinnest stripes by pressing the buttons (i.e. highest spatial frequency). To control for attention lapses an additional 7 percent of the trials served as catch trials in which a Winnie the Pooh cartoon was presented and the children had to respond to by pressing the spacebar. All included participants detected at least 85% of the catch trials correctly (range: 85–100%; median: 100%). Gabor stimuli were presented for 300 ms followed by scrambled phase masks for 300 ms. After participants responded feedback was provided. The participant could start the next trial after a self-paced duration. All participants performed the task within 25 minutes. Feedback included descriptive visual information including whether response was correct, information on winning or losing a point, and the total points earned in the current block. To study effects of reward on visual processing participants were divided into a positive reinforcement or punishment group. Participants in the reinforcement group earned one point for every correct response, those in the punishment group lost one point for every incorrect response. To further increase motivation participants were told that they played a game against another participant and received feedback on their own and the other participants score after every block. This score was computer-generated and set such that the first, third, and fourth block and hence the overall game, would be won by the participant.

### Analyses

#### Discrimination thresholds

Analyses were performed on discrimination thresholds between the two Gabor patches. Thresholds were calculated as mid-run estimates based on the method described by Wetherill & Levitt [24]. New thresholds were calculated for each of the four blocks, containing 64 trials each. Thresholds were converted to Weber fractions using the formula Δf/f where Δf is the minimal difference in spatial frequencies required for accurate discrimination and f is the reference spatial frequency (6 cpd). To detect outliers the fractions were converted to z-scores using the group average and standard deviation. Z-scores were only calculated for participants that finished the block of interest. If a participant had a z-score below -2.5 or above 2.5, it was defined as outlier and excluded from the analyses. Seven participants were excluded from the analyses (7–8 year-old: N = 2; 9–10 years: N = 3; 11–12 years: N = 1; adults: N = 1).

**Fig 2:**
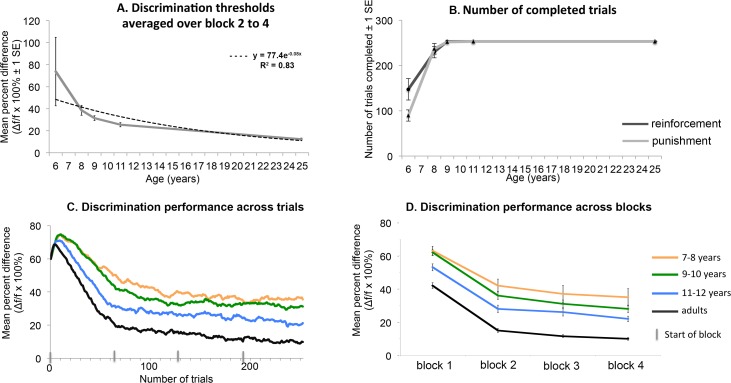
**(A)** Discrimination thresholds (Weber fractions) for per age-group averaged over block 2, 3, and 4, showing a linear decrease with age. **(B)** Number of completed trials per age-group and reward condition, showing fewer completed trials at 5–6 years, but completion of the experiment in most of the 7–12 year-olds. (**C and D)** Discrimination thresholds per age-group across trials (C) and blocks (D), showing for all age groups a decrease during the run, and a generally lower threshold with age.

Visual inspection of the data (Fig 2C) informed that thresholds could be reliably estimated in the 2^nd^ until the 4^th^ block. In contrast, in the first block no thresholds were reached yet. Therefore, analyses on discrimination thresholds only included the 2^nd^ to 4^th^ block. Furthermore, only five of the 5–6 year old children completed all four blocks. Therefore, this group is not included in discrimination threshold analyses, but is depicted in Fig 2A for purpose of visual comparison.

Discrimination thresholds were calculated using a repeated measures ANOVA with threshold as dependent variable, block (2; 3; 4) as within-subject independent variable and reward (reinforcement; punishment) and age-group (7–8; 9–10; 11–12; adult) as between-subject independent variables. Effects of block and age were further analyzed using repeated contrasts. To control whether any of the revealed effects were due to gender differences, an additional analyses included gender as between-subject independent variable. All post-hoc comparisons were Bonferroni corrected and all alpha levels were 5%.

#### Number of trials completed

To evaluate the effects of age and reward on motivation we calculated the number of completed trials. A univariate ANOVA was performed with the number of trials being the dependent variable and reward (reinforcement; punishment) and age-group (5–6; 7–8; 9–10; 11–12; adult) being the between-subject independent variables.

## Results

### Discrimination thresholds

Fig 2A presents the thresholds averaged over block 2 to 4, per age-group. Fig 2 presents the Weber-fractions across trials (2C) and blocks (2D) averaged per age-group. Please refer to S2 Fig for examples of individual results. Repeated measures ANOVA revealed an effect of age on threshold (*F*(3,116) = 14.5; *p* < .001; η^2^ = .272). Post-hoc analyses revealed no significant difference between 7 and 9 year-olds (*t*(67) = 1.5; *p* = .129; *d* = .36), a marginal decrease between 9 and 11 years (*t*(67) = 2.0; *p* = .050; *d* = .51), and a significant decrease from 11 years to adults (*t*(41.8) = 6.1; *p* < .001; *d* = 1.6). In addition, there was an effect of block on threshold (*F*(1.7,202) = 28.7; *p* < .001; η^2^ = .198). Overall, thresholds decreased with each block and fit a linear curve (*F*(1,116) = 44.7; *p* < .001). Follow-up repeated contrasts between blocks revealed significant differences between every pair of blocks (all *p* < .01) showing that higher spatial frequency discrimination thresholds decreased significantly during the run. No effect of reward condition or gender, and no interactions between any variables were revealed (all *p* > .1).

### Participation duration

Fig 2B presents the number of trials per age-group and reward condition. Univariate ANOVA revealed a significant interaction between age-group and reward on the number of trials children completed (*F*(4,155) = 3.4; *p* = .010; η^2^ = .081). Post-hoc analyses revealed that more trials were included in the reinforcement than punishment condition in the 5–6 year-olds (*t*(21) = -2.2; *p* = .042; *d* = .80) but no difference in the older groups (*p* > .1). Furthermore, there was a main effect of age-group (*F*(4,155) = 19.2; *p* = .007; η^2^ = .950), with 5–6 year-olds completing fewer trials than 7–9 year-olds (*p* < .001), and 7–9 fewer than 11–12 year-olds (*p* = .030), but no differences between the other age groups (*p* > .1). When including gender in the analyses the interaction between age-group and reward disappeared (*F*(4,149) = 1.5; *p* = .200; η^2^ = .039) and the interaction between group and gender was significant (*F*(4,149) = 3.8; *p* = .006; η^2^ = .092). Post-hoc tests revealed that in the 5–6 year old age-group, females completed more trials than males (*t*(26) = -2.9; *p* = .007; *d* = 1.2).

## Discussion

This study investigated two questions related to visual perceptual learning research of higher spatial frequency discrimination in children. The first goal was to gain a full understanding of typical development in higher spatial frequency perception. Therefore, we studied the developmental trajectory of higher spatial frequency discrimination abilities in children aged between 7 and 12 years and adults. An additional group of 5–6 year-olds were tested but excluded from the analyses because they performed too few trials to estimate thresholds. Results showed lower discrimination thresholds with age. Interestingly, discrimination had not matured at 12 years of age. Second, the study investigated effects of reward on spatial frequency discrimination and motivation. We contrasted effects of positive reinforcement (earning points for correct response) to punishment (losing points for incorrect response). The type of reward did not affect higher spatial frequency discrimination thresholds. To study whether the task would show effects of practice and be motivating for children, we investigated changes in threshold during the task (practice) and the number of trials a child completed (motivation). Discrimination thresholds decreased between block 2 and 4 in 7–12 year-olds and adults (Fig 2C and 2D). Note that performance in the first block did not lead to accurate estimation of thresholds. Therefore, this block was not included in the analyses. To evaluate motivation all age groups were included. Punishment led to completion of fewer trials than reinforcement in 5–6 year olds but reward did not affect other outcomes or age groups. Furthermore, children aged 5–6 completed only a fourth or half of the trials but 7–12 year-olds and adults completed all 254 trials (Fig 2B). The completion and decreasing thresholds indicate that for children aged 7–12 years the study presents a motivating task that can be applied in future visual perceptual learning studies.

The ongoing development in higher spatial frequency discrimination between 7 year olds and adulthood conflicts with previous behavioral studies on contrast sensitivity for higher spatial frequency gratings. Most studies show a rapid increase in sensitivity early in life with maturation at 3–6 years, although some studies reported small improvements until 9–12 years of age [2,9–12]. In addition, previous research revealed that discrimination of lower spatial frequency gratings (1 and 3 cpd) shows a rapid improvement between 5 and 7 years of age followed by a more gradual improvement. At 9 years of age children perform almost at adult levels [15]. These results cannot be generalized to higher spatial frequencies, because developmental trajectories differ between spatial frequencies [2]. The current study therefore investigated discrimination thresholds of higher spatial frequencies (6 cpd). For a valid comparison between findings, it is important to notice methodological differences that might affect performance. Whereas Patel and colleagues presented stimuli sequentially, we used simultaneous presentation. In naïve observers, such as children, simultaneous presentation could lead to lower thresholds than sequential presentation [25]. Even though stimulus sequence did not affect discrimination performance in 5-year olds [15], this factor should be considered when comparing the results between studies. Furthermore, subjects in the study of Patel could practice the task, whereas this was not the case in the current study. It should however be noted that we did not include the first block in the analyses because no threshold was reached. Consequently, this block could be considered as a practice block. Furthermore, Patel and colleagues [15] provided both positive and negative feedback, whereas the current study provided either of those. However, because we show that the type of feedback does not affect discrimination thresholds, this is unlikely to affect the results. Finally, whereas the current study presented a fixed number of trials, Patel and colleagues [15] finished the experiment when threshold could be measured with a confidence interval of 95% that the threshold was accurate within 0.1 log units. Regardless of these discrepancies, the outcome measures (i.e. Weber fractions) were equal between experiments and could thus be directly compared. Comparison between the developmental trajectories reveals that discrimination of higher spatial frequencies improves for a much longer time with large improvements even after 12 years of age, compared to almost adult-like performance at 9 years for lower spatial frequencies.

These discrepancies underline that each task measures a different aspect of spatial frequency processing. Discrimination abilities reflect spatial frequency sensitivity between different spatial frequencies for one contrast level and are thought to reflect the width of the tuning curve. Contrast sensitivity reflects the ability to perceive gratings at a minimum contrast level for one spatial frequency, possibly reflecting the amplitude of tuning curves [26–28]. The earlier maturation of contrast sensitivity compared to discrimination of higher spatial frequencies suggests that for neurons responding to these frequencies the amplitude of the tuning curve matures at an earlier age than the width. In addition the earlier maturation for discrimination of lower than higher spatial frequencies implies that the width of the tuning curve matures faster for neurons responding to lower than higher spatial frequencies.

It should be noted that even the performance of adults in the current study deviates from previous thresholds reported in adults on high spatial frequency discrimination (e.g. [29–32]). Whereas just noticeable differences of 5% or less are reported before, we find a difference of 12%. Several methodological factors differ between studies, including presentation settings and participants. Most presentation settings are unlikely to explain the discrepancies: for instance, whereas sequential stimulus presentation was used in previous studies, as opposed to parallel presentation in the current one, the presentation sequence does not affect discrimination thresholds [30]. In addition, although presentation duration is lower in the current (300 ms) than most previous studies (600 to 1500 ms or unlimited time), adults can discriminate spatial frequencies even at 16 ms [19]. However, because discrimination abilities were not measured at very low differences in [17], it remains unclear whether presentation duration affects performance. The factor that most likely explains differences between studies relates to the subjects. Previous studies often investigated discrimination in experienced subjects (for instance the authors). In contrast, naïve subjects participated in the current study. Naïve subjects perform worse on psychophysical tasks than experienced subjects [25]. As such, the difference in subjects might contribute to the discrepancies in discrimination thresholds from previous findings.

The second goal of the study was to investigate the effects of reward on higher spatial frequency discrimination and on participation duration. The previous study on spatial frequency discrimination [13] provided both positive and negative reward, and could thus not specify whether the *type* of reward affects discrimination thresholds. The current results reveal that this is not the case, which rejects the hypothesis that positive reinforcement leads to better performance than punishment. Van Duijvenvoorde and colleagues [22] observed an increased performance in cognitive tasks when children were positively rewarded. A similar performance gain is also predicted for perceptual learning tasks by a theoretical model of Roelfsema et al. [21]. The model proposes that in case of a positive reward, higher-order areas increase the strength of lower-level neurons involved in basic visual information processing. The current results indicate that the type of reward does not affect performance in children. However, it is still possible that reward in itself, in contrast to an absence of reward, improves performance. The presence of reward affects various cognitive tasks in adults [33]. Another possibility is that reward only affects performance on more complex or cognitive processes. An interesting direction for future research is to contrast positive reinforcement and punishment with an absence of reward. Furthermore, research needs to show for which modality reward specifically affects performance and learning in children.

The current results lead to recommendations and directions for future developmental research in visual perceptual learning. For children aged between 7 to 12 years the task can be applied as presented here. The participation for a long duration (254 trials in less than 30 minutes) shows that the current methods are motivating enough for children. Long participation duration is required in visual perceptual learning designs in adults but is not typically displayed in developmental literature. The task can be applied including either positive reinforcement or punishment as a reward, because using either did not affect the results. Furthermore, the task can be easily adapted to study discrimination of other spatial frequencies or contrast sensitivity. For instance, whereas high spatial frequencies drive emotion discrimination in 3 to 8 year-olds [23], lower or mid-band spatial frequencies play a crucial role in older children and adults (e.g. [5]. Discrimination of these frequencies could be investigated and possibly improved using an adapted version of the current task as well. Future research should investigate whether the decreasing thresholds across blocks also represent practice effects that remain across days. In addition, studies need to examine whether perception can be further improved in multiple sessions. Several studies showed the importance of sleep in perceptual learning [34]. This suggests that effects will be even larger if children participate in multiple sessions across days. For children aged 5 to 6 years several adaptations to the task are required. Most of these children did not complete the run due to a lack of motivation or attention. This indicates that 5–6 year olds could perform the task but require a more motivating design or multiple short sessions to participate in visual perceptual learning studies. Future studies should reveal whether adapted designs and shorter sessions successfully induce learning in this age group. Eventually, this task might be applied in clinical populations to improve visual perception. However, these populations might suffer from attentional difficulties as well, even at older ages than 5–6 years. To apply this task in clinical age-groups, it should be further adapted to capture and remain the child’s attention. For all age groups the pattern of responses in the first block suggests that participants need substantial practice in the task requirements and design in order to measure thresholds reliably. After sufficient practice, participants could start at a lower spatial frequency difference between Gabors. By starting at a spatial frequency difference that is closer to the here-reported thresholds, participants need to complete fewer trials to estimate a reliable threshold. Future perceptual learning studies, based on the current task, might benefit children with atypical vision. They could especially aid the low-level visual processing deficits such as reported in patients treated for congenital cataract [4,35].

In conclusion, the current study presents the developmental trajectory of higher spatial frequency discrimination. Specifically, discrimination improved between 7 year olds and adulthood. Future studies need to address whether discrimination can be further improved in multiple sessions before the method is optimized to correct atypical vision. Furthermore, this study describes a task that is motivating, and successfully induces improvement of spatial frequency discrimination in 7–12 year-old children. As such it presents both the developmental trajectory of higher spatial frequency discrimination and a tool for visual perceptual learning research in children.

## Supporting Information

S1 Fig Example of presented stimuli. The spatial frequency of the left Gabor (reference) is 6 cycles per degree, and of the right Gabor 4 cycles per degree when depicted at a screen resolution of 1920x1080 and viewing distance of 57 cm. Hence, the difference in spatial frequency between Gabors is 33%.
(TIF)

S2 Fig Example of individual results. Discrimination thresholds across trials per age-group. Each line shows the results of individual participants representative for the group performance.
(TIF)
