# Supplementary figures and images for: Spatial Frequency Discrimination: Effects of Age, Reward, and Practice
Source: PLoS One. 2017 Jan 30;12(1):e0169800. doi: 10.1371/journal.pone.0169800 (PMC5279743; doi:10.1371/journal.pone.0169800)

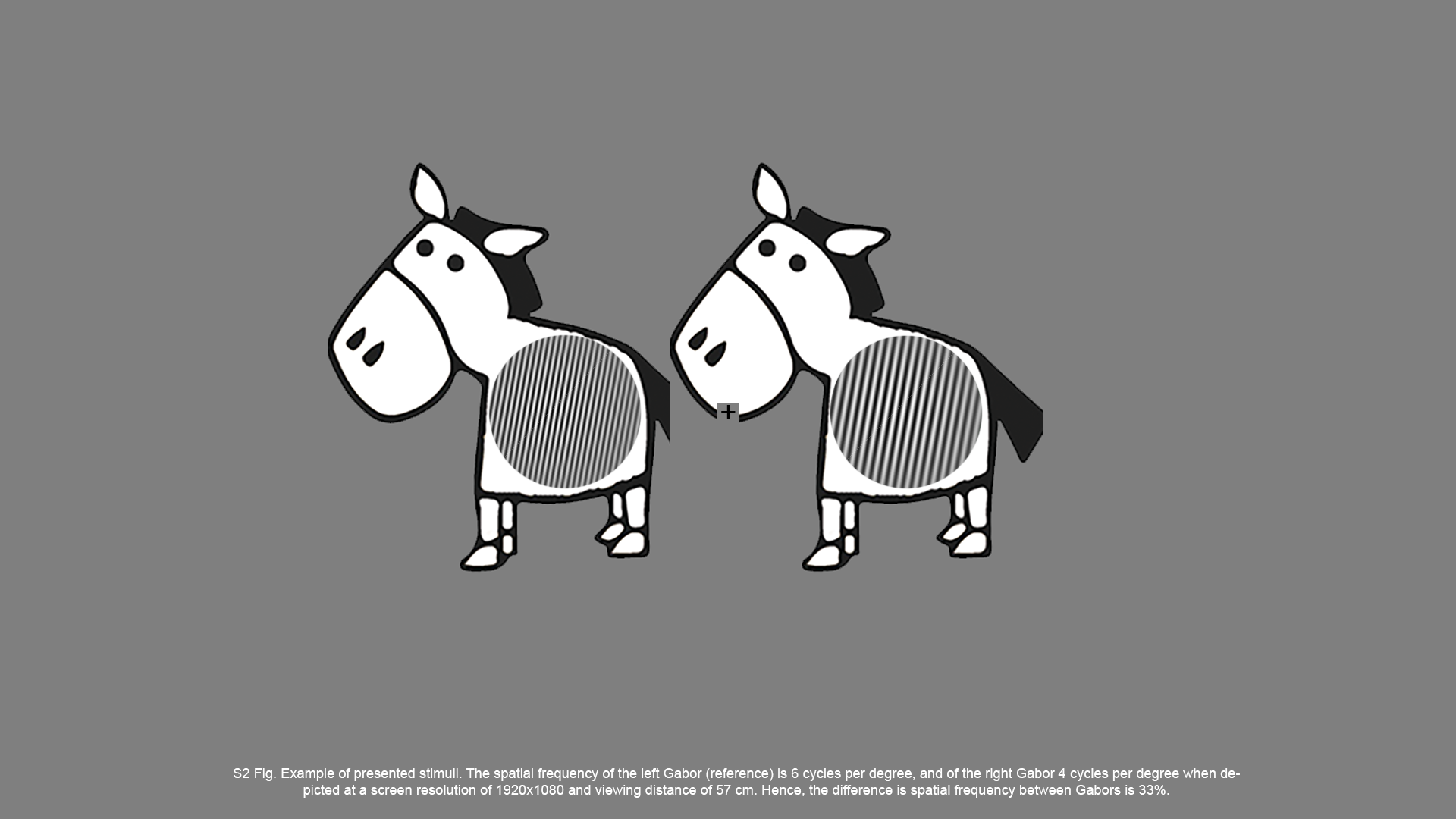

Supplement: S1 Fig — The spatial frequency of the left Gabor (reference) is 6 cycles per degree, and of the right Gabor 4 cycles per degree when depicted at a screen resolution of 1920x1080 and viewing distance of 57 cm. Hence, the difference in spatial frequency between Gabors is 33%. (TIF) [file pone.0169800.s001.tif]

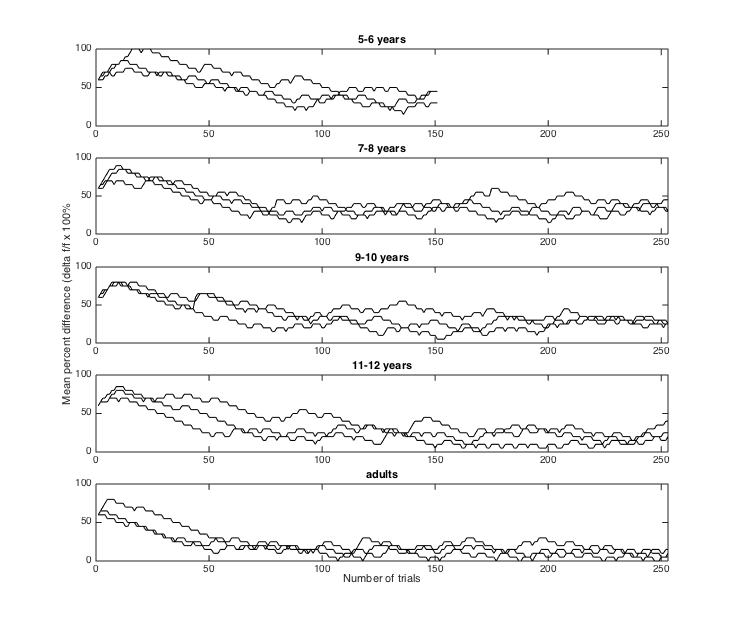

Supplement: S2 Fig — Discrimination thresholds across trials per age-group. Each line shows the results of individual participants representative for the group performance. (TIF) [file pone.0169800.s002.tif]
